# Supplementary material for: Impact and perceptions of Active Learning Classrooms on reducing sedentary behaviour and improving physical and mental health and academic indicators in children and adolescents: A scoping review
Source: PLoS One. 2025 Feb 5;20(2):e0317973. doi: 10.1371/journal.pone.0317973 (PMC11798443; doi:10.1371/journal.pone.0317973)
Supplement: S2 Table — (DOCX) [file pone.0317973.s002.docx]

**S2 Table. Summary of the search strategy**.

| **Database** | **Strategy** | **Result** |
| --- | --- | --- |
| **Pubmed** | ("classroom physical environment"[Title] OR "innovative learning environmental"[Title] OR "innovative learning spaces"[Title] OR "classroom environmental"[Title] OR "school environmental"[Title] OR "classroom-based physical activity"[Title] OR "active-permissive environment"[Title] OR "active permissive classroom"[Title] OR "classroom configurations"[Title] OR "classroom design"[Title] OR "classroom layout"[Title] OR "dynamic seating"[Title] OR "active school design"[Title] OR "sit-stand desks"[Title] OR "standing workstation"[Title] OR "standing desk"[Title] OR "stability ball"[Title] OR "bike desk"[Title] OR "flexible learning"[Title] OR "flexible learning spaces"[Title] OR "open learning spaces"[Title] OR "flexible classroom seating"[Title] OR "conventional classroom"[Title] OR "non-traditional classroom"[Title] OR "fixed classroom"[Title] OR "flexible furniture"[Title] OR furniture[Title] OR "flexible seating"[Title] OR "innovative classroom"[Title] OR "open classroom"[Title] OR "movement integration"[Title] OR "learning spaces"[Title]) AND ("movement behaviors"[Title/Abstract] OR movement[Title/Abstract] OR "sedentary behavior"[Title/Abstract] OR "physical activity"[Title/Abstract] OR "sedentary time"[Title/Abstract] OR "breaks in sitting"[Title/Abstract] OR "standing time"[Title/Abstract] OR "stepping time"[Title/Abstract] OR "steps"[Title/Abstract] OR "cardiorespiratory fitness"[Title/Abstract] OR fitness[Title/Abstract] OR adiposity[Title/Abstract] OR "fat mass"[Title/Abstract] OR "body composition"[Title/Abstract] OR obesity[Title/Abstract] OR "cardiometabolic profile"[Title/Abstract] OR cardiovas*[Title/Abstract] OR "physical health"[Title/Abstract] OR cognition[Title/Abstract] OR "executive function"[Title/Abstract] OR intelligence[Title/Abstract] OR memory[Title/Abstract] OR attention[Title/Abstract] OR "brain development"[Title/Abstract] OR "brain health"[Title/Abstract] OR "academic achievement"[Title/Abstract] OR "academic performance"[Title/Abstract] OR "academic grades"[Title/Abstract] OR "academic success"[Title/Abstract] OR "learning behaviors"[Title/Abstract] OR "classroom behaviour"[Title/Abstract] OR "on-task behavior"[Title/Abstract] OR autonomy[Title/Abstract] OR concentration[Title/Abstract] OR "behavioral engagement"[Title/Abstract] OR "cognitive engagement"[Title/Abstract] OR "health-related quality of life"[Title/Abstract] OR "quality of life"[Title/Abstract] OR "well-being"[Title/Abstract] OR "positive health"[Title/Abstract] OR "psychological health"[Title/Abstract] OR "mental health"[Title/Abstract] OR perceptions[Title/Abstract] OR barriers[Title/Abstract] OR limitations[Title/Abstract] OR attitudes[Title/Abstract] OR consequences[Title/Abstract] OR feasibility[Title/Abstract] OR "implementation strategies"[Title/Abstract] OR effects[Title/Abstract] OR impact[Title/Abstract]) | 255 |
| **Scopus** | TITLE ("classroom physical environment” OR "innovative learning environmental” OR "innovative learning spaces” OR "classroom environmental” OR "school environmental” OR "classroom-based physical activity" OR "active-permissive environment” OR "active permissive classroom” OR "classroom configurations” OR "classroom design” OR "classroom layout" OR "dynamic seating” OR "active school design" OR "sit-stand desks" OR "standing workstation" OR "standing desk" OR "stability ball" OR "bike desk" OR "flexible learning" OR "flexible learning spaces" OR "open learning spaces" OR “flexible classroom seating" OR “conventional classroom" OR “non-traditional classroom" OR "fixed classroom" OR "flexible furniture" OR furniture OR "flexible seating” OR "innovative classroom" OR "open classroom" OR "movement integration” OR "learning spaces”) AND TITLE (( "movement behaviors” OR movement OR "sedentary behavior” OR "physical activity” OR "sedentary time” OR "breaks in sitting” OR "standing time” OR "stepping time” OR "steps" OR "cardiorespiratory fitness” OR fitness OR adiposity OR "fat mass” OR "body composition” OR obesity OR "cardiometabolic profile” OR cardiovas* OR "physical health” OR cognition OR "executive function" OR intelligence OR memory OR attention OR "brain development” OR "brain health" OR "academic achievement” OR "academic performance” OR "academic grades” OR "academic success" OR "learning behaviors" OR "classroom behaviour" OR "on-task behavior” OR autonomy OR concentration OR "behavioral engagement” OR "cognitive engagement" OR "health-related quality of life” OR "quality of life” OR "well-being” OR "positive health” OR "psychological health” OR "mental health” OR perceptions OR barriers OR limitations OR attitudes OR consequences OR feasibility OR "implementation strategies" OR effects OR impact) | 644 |
| **ERIC** | TI ( ("classroom physical environment” OR "innovative learning environmental” OR "innovative learning spaces” OR "classroom environmental” OR "school environmental” OR "classroom-based physical activity" OR "active-permissive environment” OR "active permissive classroom” OR "classroom configurations” OR "classroom design” OR "classroom layout" OR "dynamic seating” OR "active school design" OR "sit-stand desks" OR "standing workstation" OR "standing desk" OR "stability ball" OR "bike desk" OR "flexible learning" OR "flexible learning spaces" OR "open learning spaces" OR “flexible classroom seating" OR “conventional classroom" OR “non-traditional classroom" OR "fixed classroom" OR "flexible furniture" OR furniture OR "flexible seating” OR "innovative classroom" OR "open classroom" OR "movement integration” OR "learning spaces”) ) AND AB ( ("movement behaviors” OR movement OR "sedentary behavior” OR "physical activity” OR "sedentary time” OR "breaks in sitting” OR "standing time” OR "stepping time” OR "steps" OR "cardiorespiratory fitness” OR fitness OR adiposity OR "fat mass” OR "body composition” OR obesity OR "cardiometabolic profile” OR cardiovas* OR "physical health” OR cognition OR "executive function" OR intelligence OR memory OR attention OR "brain development” OR "brain health" OR "academic achievement” OR "academic performance” OR "academic grades” OR "academic success" OR "learning behaviors" OR "classroom behaviour" OR "on-task behavior” OR autonomy OR concentration OR "behavioral engagement” OR "cognitive engagement" OR "health-related quality of life” OR "quality of life” OR "well-being” OR "positive health” OR "psychological health” OR "mental health” OR perceptions OR barriers OR limitations OR attitudes OR consequences OR feasibility OR "implementation strategies" OR effects OR impact) ) | 252 |
| **ProQuest Education** | TI ( "classroom physical environment” OR "innovative learning environmental” OR "innovative learning spaces” OR "classroom environmental” OR "school environmental” OR "classroom-based physical activity" OR "active-permissive environment” OR "active permissive classroom” OR "classroom configurations” OR "classroom design” OR "classroom layout" OR "dynamic seating” OR "active school design" OR "sit-stand desks" OR "standing workstation" OR "standing desk" OR "stability ball" OR "bike desk" OR "flexible learning" OR "flexible learning spaces" OR "open learning spaces" OR “flexible classroom seating" OR “conventional classroom" OR “non-traditional classroom" OR "fixed classroom" OR "flexible furniture" OR furniture OR "flexible seating” OR "innovative classroom" OR "open classroom" OR "movement integration” OR "learning spaces” ) AND AB ( ("movement behaviors” OR movement OR "sedentary behavior” OR "physical activity” OR "sedentary time” OR "breaks in sitting” OR "standing time” OR "stepping time” OR "steps" OR "cardiorespiratory fitness” OR fitness OR adiposity OR "fat mass” OR "body composition” OR obesity OR "cardiometabolic profile” OR cardiovas* OR "physical health” OR cognition OR "executive function" OR intelligence OR memory OR attention OR "brain development” OR "brain health" OR "academic achievement” OR "academic performance” OR "academic grades” OR "academic success" OR "learning behaviors" OR "classroom behaviour" OR "on-task behavior” OR autonomy OR concentration OR "behavioral engagement” OR "cognitive engagement" OR "health-related quality of life” OR "quality of life” OR "well-being” OR "positive health” OR "psychological health” OR "mental health” OR perceptions OR barriers OR limitations OR attitudes OR consequences OR feasibility OR "implementation strategies" OR effects OR impact) ) | 201 |
| **Total** |  | **1352** |
|  |  |  |
