# Supplementary material for: Impact and perceptions of Active Learning Classrooms on reducing sedentary behaviour and improving physical and mental health and academic indicators in children and adolescents: A scoping review
Source: PLoS One. 2025 Feb 5;20(2):e0317973. doi: 10.1371/journal.pone.0317973 (PMC11798443; doi:10.1371/journal.pone.0317973)
Supplement: S3 Table — (DOCX) [file pone.0317973.s003.docx]

| **Reference** | **URL** | **Reason for exclusion** |
| --- | --- | --- |
| Bradbeer C, et al; 2017 | <https://doaj.org/article/d093b54782e54b9bb3a5696585526399> | Not fit the definition of Active Learning Classroom |
| Carvalho L, et al; 2020 | <https://link.springer.com/article/10.1007/s10984-020-09311-4> | Not examine study outcomes |
| Donovan, L et al; 2014 | <https://journals.sagepub.com/doi/10.2190/EC.50.2.a> | Not fit the definition of Active Learning Classroom |
| Everat J, et al; 2018 | <https://www.tandfonline.com/doi/abs/10.1080/03004279.2018.1538256> | Not examine study outcomes |
| Fletcher J & Everatt J, 2021 | <https://link.springer.com/article/10.1007/s40841-021-00195-3> | Focused on higher education (collages) |
| Fletcher J, et al; 2023 | <https://link.springer.com/article/10.1007/s40841-023-00280-9> | Not examine study outcomes |
| Kariippanon KE, et al; 2019 | <https://link.springer.com/article/10.1007/s10833-019-09364-0> | Not examine study outcomes |
| Magen-Naga N & Steinberger P; 2017 | <https://link.springer.com/article/10.1007/s10984-017-9232-2#:~:text=According%20to%20students'%20perceptions%2C%20the,in%20both%20the%20actual%20and> | Not fit the definition of Active Learning Classroom |
| Morris JE & Imms W, 2022 | <https://iulresearch.iuline.it/index.php/IUL-RES/article/view/295> | Not examine study outcomes |
| Starkey L, et al; 2021 | <https://link.springer.com/article/10.1007/s40841-020-00187-9> | Not examine study outcomes |
| Zhanga, Y et al; 2023 | <https://www.tandfonline.com/doi/full/10.1080/13467581.2023.2260852> | Not fit the definition of Active Learning Classroom |

**S3 Table. Studies excluded after full text read with the reasons for exclusion.**
