# Supplementary material for: Impact and perceptions of Active Learning Classrooms on reducing sedentary behaviour and improving physical and mental health and academic indicators in children and adolescents: A scoping review
Source: PLoS One. 2025 Feb 5;20(2):e0317973. doi: 10.1371/journal.pone.0317973 (PMC11798443; doi:10.1371/journal.pone.0317973)
Supplement: Attachment — Submitted filename: Response to Reviewers.docx [file pone.0317973.s004.docx]

Heather Macdonald, Ph.D

Academic Editor

PLOS ONE

Dear Editor,

Please find enclosed a revised version of our manuscript, “Impact and perceptions of Active Learning Classrooms on reducing sedentary behaviour and improving physical and mental health and academic indicators in children and adolescents: a scoping review” (PONE-D-24-40271). We would like to thank you for giving us the opportunity to revise and improve our manuscript; we also thank the reviewers for the thoughtful and constructive comments. We have considered all the suggestions and have incorporated them into the revised manuscript ('Revised Manuscript with Track Changes'), and as a result, we believe our manuscript is stronger. An itemized point-by-point response to the academic editor and reviewers’ comments is shown below.

Thank you for your attention to this matter.

**Response to Journal Requirements:**

[When submitting your revision, we need you to address these additional requirements.

1. Please ensure that your manuscript meets PLOS ONE's style requirements, including those for file naming. The PLOS ONE style templates can be found at https://journals.plos.org/plosone/s/file?id=wjVg/PLOSOne_formatting_sample_main_body.pdf and](http://track.editorialmanager.com/CL0/file:%2F%2F%2FC:%2FUsers%2Fe754871%2FDesktop%2FPRTC%2FPRTC%25202024%2FOctober%25202024%2FOctober%252025,%25202024%2FPONE-D-24-40271%2FJournal%2520requirements:%2520%250b%250bWhen%2520submitting%2520your%2520revision,%2520we%2520need%2520you%2520to%2520address%2520these%2520additional%2520requirements.%2520%250b%250b1.%2520Please%2520ensure%2520that%2520your%2520manuscript%2520meets%2520PLOS%2520ONE's%2520style%2520requirements,%2520including%2520those%2520for%2520file%2520naming.%2520The%2520PLOS%2520ONE%2520style%2520templates%2520can%2520be%2520found%2520at%2520https:%2Fjournals.plos.org%2Fplosone%2Fs%2Ffile%253fid=wjVg%2FPLOSOne_formatting_sample_main_body.pdf%2520and/1/010f0192c4b9ea86-2c90c9ef-f76d-4dfd-ba78-bc10ac16f96a-000000/VEE1BRgwrj5yksgCCXV-Ag0_wFJ17LXZH_zt31jAFKs=182) [https://journals.plos.org/plosone/s/file?id=ba62/PLOSOne_formatting_sample_title_authors_affiliations.pdf](https://track.editorialmanager.com/CL0/https:%2F%2Fjournals.plos.org%2Fplosone%2Fs%2Ffile%3Fid=ba62%2FPLOSOne_formatting_sample_title_authors_affiliations.pdf/1/010f0192c4b9ea86-2c90c9ef-f76d-4dfd-ba78-bc10ac16f96a-000000/CXW-tffXVpK-EKROHx19xNgx7_YfsFMmaDK3uGeDEO0=182)

**Author’s response:** Thank you for the recommendation. We have carefully revised the format to meet the PlosOne guidelines.

2. We note that the grant information you provided in the ‘Funding Information’ and ‘Financial Disclosure’ sections do not match. When you resubmit, please ensure that you provide the correct grant numbers for the awards you received for your study in the ‘Funding Information’ section.

**Author’s response:** Thank you for your comment. The information is now the same in both sections. It can be read as follows:

*This review was conducted while Mairea Sánchez-López was benefiting from a research grant awarded by the Spanish Ministry of Universities (Senior Mobility Grants ‘Salvador Madariaga 2022’, reference number: PRX22/00245). The founders had no role in study design, data collection and analysis, decision to publish, or preparation of the manuscript.*

3. Thank you for stating the following financial disclosure: “This review was conducted while Mairea Sánchez-López was benefiting from a research grant awarded by the Spanish Ministry of Universities (Senior Mobility Grants ‘Salvador Madariaga 2022’, reference number: PRX22/00245).”

Please state what role the funders took in the study. If the funders had no role, please state: "The funders had no role in study design, data collection and analysis, decision to publish, or preparation of the manuscript." If this statement is not correct you must amend it as needed. Please include this amended Role of Funder statement in your cover letter; we will change the online submission form on your behalf.

**Author’s response:** Thank you for your comment. We have added the Rol of Funder in the ‘Funding Information’ section and in the ‘Financial Disclosure section’. In addition, we have updated the cover letter with this information.

*This review was conducted while Mairea Sánchez-López was benefiting from a research grant awarded by the Spanish Ministry of Universities (Senior Mobility Grants ‘Salvador Madariaga 2022’, reference number: PRX22/00245). The founders had no role in study design, data collection and analysis, decision to publish, or preparation of the manuscript.*

4. We note that your Data Availability Statement is currently as follows: “All relevant data are within the manuscript and in Supporting Information files.”

Please confirm at this time whether or not your submission contains all raw data required to replicate the results of your study. Authors must share the “minimal data set” for their submission. PLOS defines the minimal data set to consist of the data required to replicate all study findings reported in the article, as well as related metadata and methods (<https://journals.plos.org/plosone/s/data-availability#loc-minimal-data-set-definition>). For example, authors should submit the following data: - The values behind the means, standard deviations and other measures reported; - The values used to build graphs; - The points extracted from images for analysis. Authors do not need to submit their entire data set if only a portion of the data was used in the reported study. If your submission does not contain these data, please either upload them as Supporting Information files or deposit them to a stable, public repository and provide us with the relevant URLs, DOIs, or accession numbers. For a list of recommended repositories, please see <https://journals.plos.org/plosone/s/recommended-repositories>. If there are ethical or legal restrictions on sharing a de-identified data set, please explain them in detail (e.g., data contain potentially sensitive information, data are owned by a third-party organization, etc.) and who has imposed them (e.g., an ethics committee). Please also provide contact information for a data access committee, ethics committee, or other institutional body to which data requests may be sent. If data are owned by a third party, please indicate how others may request data access.

**Author’s response:** We state that all relevant data for replicating this study are within the manuscript and in Supporting Information files.

5. As required by our policy on Data Availability, please ensure your manuscript or supplementary information includes the following: A numbered table of all studies identified in the literature search, including those that were excluded from the analyses. For every excluded study, the table should list the reason(s) for exclusion. If any of the included studies are unpublished, include a link (URL) to the primary source or detailed information about how the content can be accessed. A table of all data extracted from the primary research sources for the systematic review and/or meta-analysis. The table must include the following information for each study: Name of data extractors and date of data extraction Confirmation that the study was eligible to be included in the review. All data extracted from each study for the reported systematic review and/or meta-analysis that would be needed to replicate your analyses. If data or supporting information were obtained from another source (e.g. correspondence with the author of the original research article), please provide the source of data and dates on which the data/information were obtained by your research group. If applicable for your analysis, a table showing the completed risk of bias and quality/certainty assessments for each study or outcome. Please ensure this is provided for each domain or parameter assessed. For example, if you used the Cochrane risk-of-bias tool for randomized trials, provide answers to each of the signalling questions for each study. If you used GRADE to assess certainty of evidence, provide judgements about each of the quality of evidence factor. This should be provided for each outcome. An explanation of how missing data were handled. This information can be included in the main text, supplementary information, or relevant data repository. Please note that providing these underlying data is a requirement for publication in this journal, and if these data are not provided your manuscript might be rejected.

**Author’s response:** Thank you for your comment, we have added in supplementary information (S2 Table) a table explaining the reasons for exclusion of those studies that appeared to meet the inclusion criteria but were excluded after a full reading of the text.

6. Please review your reference list to ensure that it is complete and correct. If you have cited papers that have been retracted, please include the rationale for doing so in the manuscript text, or remove these references and replace them with relevant current references. Any changes to the reference list should be mentioned in the rebuttal letter that accompanies your revised manuscript. If you need to cite a retracted article, indicate the article’s retracted status in the References list and also include a citation and full reference for the retraction notice.

**Author’s response:** Done. Thank you.

**Additional Editor Comments:**

The manuscript is well-written and provides a thorough review of the literature related to active learning classrooms. In addition to the Reviewers' comments, please consider these additional comments:

1.Title: Should Active Learning Classroom be made plural here as per the rest of the manuscript?

**Author’s response:** We agree. We have changed the title to make it plural. Thank you.

2. Lines 94-97: I find the wording here awkward - please revise. It seems that the review addressed the impact of ALCs on pedagogical strategies, but the objective related to perceptions does not fit with the objective related to the impact of ALCs on... (i.e., it doesn't sense to say "...the impact of ALCs on perceptions of the school community about ALCs...). Perhaps a 2nd objective is needed that focuses on implementation/perceptions of ALCs. In turn, in the results, perceptions would not fall under the subheading related to Impact of ALC interventions.

**Author’s response:** Thank you for the comment. We have reformulated the objectives as follows:

*The objectives of this scoping review were to: (i) synthesise the existing literature on the impact of ALCs on reducing sedentary behaviour, increasing PA, well-being and academic indicators in children and adolescents; and (ii) describe the educational community's perceptions and teaching practices used in these learning spaces.*

In addition, in the Results section we have added the following subheading to separate the results corresponding to objective (ii):

*Community's perceptions and teaching practices in Active Learning Classrooms*

4. Lines 115-124: The wording of this list could be improved, particularly on lines 120-121 - reported at least one "physical" and "mental" doesn't make sense. Also, the last part of the sentence regarding methods could be a separate sentence.

**Author’s response**: Thank you. We have reworded the paragraph to make the wording clearer:

-In Material and Methods- Eligibility and Exclusion Criteria:

*Studies were included if they met the following criteria:*

*(i) were conducted in pre-school, primary, or secondary school classrooms (age range: 3–18 years) within standard classroom settings;*

*(ii) reported on the impact of ALCs in reducing sedentary behaviour and improving the physical and mental health and academic indicators of students, with at least one variable reported in the following categories: movement behaviour (e.g., physical activity, sedentary time, standing, and sitting time); physical health (e.g., adiposity, pain); mental health (e.g., psychological well-being, stress); or academic indicators (e.g., grades, cognition, engagement). In this review an ALC refers to educational environments designed to facilitate not only physical movement within the classroom, but also active student-centred participation, even if unplanned. These spaces often include flexible and non-traditional furniture arrangements that support a variety of collaborative and individualised work configurations.*

*(iii) examined the perceptions of students, teachers, families, or staff regarding ALCs, including aspects such as acceptability, perceived barriers and facilitators, or the teaching practices employed in these learning spaces.*

*Quantitative, qualitative and mixed methods study design were included.*

5. Line 125: Change to "Studies were included if they: focused on higher education..." and then ensure that the text matches for each exclusion criteria (i.e., change "include" to "included" on Line 130.

**Author’s response**: Thank you for your appreciation. We have modified the paragraph as follows:

-In Material and Methods- Eligibility and Exclusion Criteria.

*Studies were excluded if they met any of the following criteria:*

*(i) focused on higher education (e.g., universities) or mixed groups (e.g., school-aged children or adolescents and adults);*

*(ii) involved special population groups (e.g., children with clinically significant behavioral disorders, such as attention difficulties, or children with overweight/obesity);*

*(iii) investigated aspects of the built environment in the classroom, including factors such as temperature, light, color, noise, or overall environmental quality;*

*(iv) included interventions that did not align with the definition of ALC established in this review, as well as those solely involving the replacement of traditional desks with standing desks, fit balls, or pedal desks. These interventions were excluded because they primarily represent changes in posture during classes and do not necessarily modify classroom configuration or teaching-learning dynamics, even if such changes occur incidentally;*

*(v) full-text access was unavailable, and the authors did not respond to requests for additional data or the complete text;*

*(vi) were categorized as text or opinion articles, conference abstracts, doctoral theses, dissertations, or review articles.*

6. Line 136: Consider starting a new sentence with "Studies were screened..."

**Author’s response:** Done. Thank you.

7. Lines 154-156: Consider changing the language here to say that 1084 publications were screened, and then 18 eligible publications were included. Please also clarify if the 18 publications represented 18 independent studies.

**Author’s response:** Thank you for your suggestion. We have rephrased the paragraph as follows:

*The electronic search identified 1352 references. After removal duplicates, 1004 studies were screened, and then 19 eligible publications were included [15–21,27–38], which represented 19 independent studies. The search results and the selection process of studies are presented in a flow chart (Fig 1). Studies excluded after reading the full text with reasons for exclusion are shown in supplementary information (S2 Table).*

8. Line 253: Change Active Learning Space to Active Learning Classroom.

**Author’s response:** Done. Thank you.

9. Line 318: Feasibility is mentioned here but not in the methods and I don't see it listed in the search terms in the supplementary table. It would be interesting to know how many studies reported feasibility indicators such as adherence. Similarly, in the last paragraph of the paper, the authors mention implementation strategies, yet strategies are only mentioned once in the discussion and implementation/implementation strategies were not included in the search strategy. This would be valuable information to consider.

**Author’s response:** Thank you for your valuable comments. We have repeated the search by including these two terms (feasibility and ‘implementation strategies’) in the search strategy. We have located one more article that meets the inclusion criteria. The reference has also been added to the References section.

Kariippanon KE, Cliff DP, Lancaster SL, Okely AD, Parrish AM. Perceived interplay between flexible learning spaces and teaching, learning and student wellbeing. Learning Environ Res. 2018; 21, 301–320. Doi: 10.1007/s10984-017-9254-9

Consequently, we have modified table S1 (search strategy) and the flow chart (Fig 1). And several parts of the manuscript. Additionally, we have modified the following paragraph in the Results section and added the following one in Future Directions (in Discussion) to better address the topic:

-Characteristics of Active Learning Classroom interventions (in Results)

*In one study, teachers received guidance on how to utilize these spaces effectively [27], while two other studies provided recommendations on the most appropriate design and teaching strategies for these environments [16, 21]. Additionally, one study highlighted that the design of these spaces supported three distinct modalities of learning: teacher-centered, learner-centered, and informal approaches.*

-In Future Directions (in Discussion)

*Finally, none of the studies addressed adherence to interventions, and only four of the 19 included studies reported strategies for implementing ACL. Given the importance of these factors for scalability, future research should focus on addressing these gaps.*

**5. Review Comments to the Author**

**Reviewer #1:**

Dear Authors,
Overall, the manuscript is informative and reviews research on Active Learning Classroom (ALC) and various behavioural, health and academic indicators in children and adolescents in the school setting. The comments and questions below highlight points that need further attention and work.

Comments:
1) Introduction, page 1. Although ALC lacks a universally accepted definition, I recommend explaining ‘active learning’ somewhat more clearly (line 68). Does the term only refer to learning that occurs while students are moving? Or is it something more than that?

**Author’s response:** We thank the reviewer for this valuable comment, which has helped us to clarify this point. As noted in the introduction, there is no universal definition for this term; however, most studies converge on the understanding that open, flexible or active learning environments differ from traditional classroom settings (e.g. rows of desks facing forward). Instead, these environments are characterised by flexible furniture and spatial arrangements that support movement within classroom while encouraging student-centred learning. Following the reviewer's suggestion, we have redrafted the paragraph to improve the definition of ACLs:

-In Introduction section:

*Although it is challenging to find a universally accepted definition for this term, in this review, ALC refers to educational environments designed to facilitate not only physical movement within the classroom, but also active student-centred participation, even if unplanned. These spaces typically include flexible, non-traditional furniture arrangements that support various collaborative and individualized working configurations, encouraging students to take an active role in their learning [14].*

-In Material and Methods- Eligibility and Exclusion Criteria:

*Studies were included if they met the following criteria:*

*(i) were conducted in pre-school, primary, or secondary school classrooms (age range: 3–18 years) within standard classroom settings;*

*(ii) reported on the impact of ALCs in reducing sedentary behaviour and improving the physical and mental health and academic indicators of students, with at least one variable reported in the following categories: movement behaviour (e.g., physical activity, sedentary time, standing, and sitting time); physical health (e.g., adiposity, pain); mental health (e.g., psychological well-being, stress); or academic indicators (e.g., grades, cognition, engagement). In this review an ALC refers to educational environments designed to facilitate not only physical movement within the classroom, but also active student-centred participation, even if unplanned. These spaces often include flexible and non-traditional furniture arrangements that support a variety of collaborative and individualised work configurations.*

*(iii) examined the perceptions of students, teachers, families, or staff regarding ALCs, including aspects such as acceptability, perceived barriers and facilitators, or the teaching practices employed in these learning spaces.*

*Quantitative, qualitative and mixed methods study design were included.*

-In Material and Methods- Eligibility and Exclusion Criteria.

*Studies were excluded if they met any of the following criteria:*

*(i) focused on higher education (e.g., universities) or mixed groups (e.g., school-aged children or adolescents and adults);*

*(ii) involved special population groups (e.g., children with clinically significant behavioral disorders, such as attention difficulties, or children with overweight/obesity);*

*(iii) investigated aspects of the built environment in the classroom, including factors such as temperature, light, color, noise, or overall environmental quality;*

*(iv) included interventions that did not align with the definition of ALC established in this review, as well as those solely involving the replacement of traditional desks with standing desks, fit balls, or pedal desks. These interventions were excluded because they primarily represent changes in posture during classes and do not necessarily modify classroom configuration or teaching-learning dynamics, even if such changes occur incidentally;*

*(v) full-text access was unavailable, and the authors did not respond to requests for additional data or the complete text;*

*(vi) were categorized as text or opinion articles, conference abstracts, doctoral theses, dissertations, or review articles.*

2) Material and Methods, page 5. You write that the search was limited to studies published in English. Later in the paragraph, you write that you also search for studies in Spanish. What is the conclusion from this? Did you not find any studies published in Spanish? Please explain or correct. Now it seems a bit contradictory.

**Author’s response:** The reviewer is right. The search was restricted to peer-reviewed articles published in English. We have corrected the error. We apologize for the inconvenience.

3) Material and Methods, page 7. The Data analysis and synthesis lack clarity. If included, please briefly explain how the data analysis was conducted. In addition, it is better to only refer to the PRISMA flow chart at the beginning of the Results section.

**Author’s response:** We appreciate the reviewer's comment. To clarify how data analysis and synthesis were conducted in this review, we have redrafted the paragraph:

*A narrative synthesis was conducted to summarize the results, and the main findings are presented in evidence tables. Additionally, we performed a Word Cloud figure to provide a visual representation of text in which words are displayed in different sizes and colours according to their frequency in the dataset. The words that appear in larger sizes and brighter colours represent the terms that are repeated in the studies to define the ALCs.*

4) Discussion, page 27. You suggest using objectively measured academic achievement as outcome measures. Please provide suggestions for some measures. Are you thinking of subject scores and grades or other measures? May be of importance to guide future research on ALC.

**Author’s response:** Thanks to the reviewer, we have specified what the reviewer commented.

Therefore, it is crucial that the future longitudinal and experimental studies would include validated measures of cognition such as executive function cognition and objectively measured academic achievement *(e.g. grades in different curriculum areas)* as outcome measures to provide direct evidence of their impact on academic indicators

5) Strengths and Limitations, page 28. It is positive that you describe the non-unanimous definitions of ALC as a limitation. However, I think that the actual heterogeneity in ALC interventions and activities also is a challenge when comparing different studies. Please add this issue when describing the possibility of recommending the design and use of classrooms.

**Author’s response**: We agree with the reviewer, thus we have added this relevant question.

*Another imitation of this review lies in the lack of a universally accepted definition of ALC. As a result, the interventions and activities conducted in these spaces are highly heterogeneous, making it challenging to compare findings across studies. This heterogeneity also limits our ability to propose a standardized approach to the design and use of such classrooms*.

6) Table 1. Please review the right column describing ‘Intervention and comparator’. For example, the description of the Attai et al. study is not complete. Report duration in a consistent way. For example, add to the Byers et al. study. Also, the Imms and Byers study is misplaced in alphabetical order. Please list the abbreviations at the end in alphabetic or any other logical order. Now the abbreviations are in adhoc order. Please correct typos ‘range’.

**Author’s response**: We appreciate the reviewer's comment. We have revised table 1 carefully to correct the errors commented by the reviewer. We have listed the abbreviations at the end of the table in alphabetical order.

7) Table 2. The Imms and Byers study is misplaced in alphabetical order. Please list the abbreviations at the end in alphabetic or any other logical order. For example, in alphabetic order, sorted by the Outcome and Main results categories. Correct typos. For example, the abbreviation IG is written as GI several times.

**Author’s response**: We appreciate the reviewer's comment. We have revised table 2 carefully to correct the errors commented by the reviewer. We have listed the abbreviations at the end of the table in alphabetical order.

I have no concerns about dual publication, research ethics, or publication ethics.

**Author’s response**: The reviewer’s comments and, in general, his/her involvement in the review process are highly appreciated.

**Reviewer #2:**

This scoping review addresses an important and timely topic in the field of physical activity and education for children and adolescents (3-18 years). The authors aim to synthesize the available literature on the impact of Active Learning Classrooms (ALCs) on reducing sedentary behavior, increasing physical activity, and improving physical health, mental health, and academic indicators in school-aged children. Additionally, they explore the perceptions of the educational community and teaching practices associated with these learning spaces.

The review followed a rigorous methodology, adhering to the Joanna Briggs Methods and PRISMA guidelines for scoping reviews. The authors conducted a comprehensive search across multiple databases, including MEDLINE, ERIC, SCOPUS, and ProQuest Education, focusing on peer-reviewed studies published in English up to 2023.

This scoping review makes a significant contribution to the field by synthesizing the current state of knowledge on ALCs and their impacts on children's health and learning. Despite the heterogeneity of interventions and some methodological limitations, the findings suggest that ALCs may offer a promising approach to reducing sedentary behavior and improving various health and educational outcomes for children and adolescents.

**Author’s response**: We appreciate the reviewer's comment.

Minor Revisions Suggested:
1. Definition of Active Learning Classrooms (ALCs)
The review would benefit from a clearer definition of ALCs in the introduction or methodology section. While the characteristics are well-described in the results, a more explicit explanation of what constitutes an ALC during the screening process would enhance clarity. The review acknowledges the heterogeneity of ALC interventions, which is appropriate for a scoping review. However, a more detailed description of the range of interventions included would provide valuable context.

***Author’s response****:* We thank the reviewer for this valuable comment, which has helped us to clarify this point. As noted in the introduction, there is no universal definition for this term; however, most studies converge on the understanding that open, flexible or active learning environments differ from traditional classroom settings (e.g. rows of desks facing forward). Instead, these environments are characterised by flexible furniture and spatial arrangements that support movement within classroom while encouraging student-centred learning. Following the reviewer's suggestion, we have redrafted the paragraph to improve the definition of ACLs:

-In Introduction section:

*Although it is challenging to find a universally accepted definition for this term, in this review, ALC refers to educational environments designed to facilitate not only physical movement within the classroom, but also active student-centred participation, even if unplanned. These spaces typically include flexible, non-traditional furniture arrangements that support various collaborative and individualized working configurations, encouraging students to take an active role in their learning [14].*

-In Material and Methods- Eligibility and Exclusion Criteria*:*

*Studies were included if they met the following criteria:*

*(i) were conducted in pre-school, primary, or secondary school classrooms (age range: 3–18 years) within standard classroom settings;*

*(ii) reported on the impact of ALCs in reducing sedentary behaviour and improving the physical and mental health and academic indicators of students, with at least one variable reported in the following categories: movement behaviour (e.g., physical activity, sedentary time, standing, and sitting time); physical health (e.g., adiposity, pain); mental health (e.g., psychological well-being, stress); or academic indicators (e.g., grades, cognition, engagement). In this review an ALC refers to educational environments designed to facilitate not only physical movement within the classroom, but also active student-centred participation, even if unplanned. These spaces often include flexible and non-traditional furniture arrangements that support a variety of collaborative and individualised work configurations.*

*(iii) examined the perceptions of students, teachers, families, or staff regarding ALCs, including aspects such as acceptability, perceived barriers and facilitators, or the teaching practices employed in these learning spaces.*

*Quantitative, qualitative and mixed methods study design were included.*

-In Material and Methods- Eligibility and Exclusion Criteria.

*Studies were excluded if they met any of the following criteria:*

*(i) focused on higher education (e.g., universities) or mixed groups (e.g., school-aged children or adolescents and adults);*

*(ii) involved special population groups (e.g., children with clinically significant behavioral disorders, such as attention difficulties, or children with overweight/obesity);*

*(iii) investigated aspects of the built environment in the classroom, including factors such as temperature, light, color, noise, or overall environmental quality;*

*(iv) included interventions that did not align with the definition of ALC established in this review, as well as those solely involving the replacement of traditional desks with standing desks, fit balls, or pedal desks. These interventions were excluded because they primarily represent changes in posture during classes and do not necessarily modify classroom configuration or teaching-learning dynamics, even if such changes occur incidentally;*

*(v) full-text access was unavailable, and the authors did not respond to requests for additional data or the complete text;*

*(vi) were categorized as text or opinion articles, conference abstracts, doctoral theses, dissertations, or review articles.*

2. Word Cloud Methodology: The pertinence of the word cloud and its methodology should be better explained, particularly in how it addresses the perceptions of the school community about ALCs and teaching practices in these spaces.

**Author’s response**: As the reviewer suggests, we have better explained the relevance of the word clouds in the manuscript.

*... Additionally, we performed a Word Cloud figure to provide a visual representation of text in which words are displayed in different sizes and colours according to their frequency in the dataset. The words that appear in larger sizes and brighter colours represent the terms that are repeated in the studies to define the ALCs.*

3. Technical Jargon:
Define 'MVPA' (Moderate to Vigorous Physical Activity) and 'LPA' (Light Physical Activity) when first introduced in the text, ideally. "Accelerometry" should also be briefly described for readers unfamiliar with the term. in the introduction alongside the concept of sedentary behavior.

**Author’s response**: Thanks to the reviewer for the comments. We have defined the abbreviations MVPA and LPA the first time they appear and added the following paragraph to define what an accelerometer is.

*Sedentary behaviour can be assessed using report-based methods, such as questionnaires, and device-based methods, such as accelerometers, which are motion sensors that record signals of the magnitude and frequency of body acceleration and can be used to measure the frequency, intensity and duration of movement*.

4. Resluts Presentation:
In the section on Movement Behaviors and Physical Health, it is stated, 'In contrast, a decrease was reported in LPA, total PA, and MVPA in students attending ALCs [31,32,34],' but the results table does not present data on LPA.

**Author’s response**: We thank the reviewer for the appreciation, we have placed the references correctly to match the information in the table.

*Schoolchildren attending in ALCs accumulated lower levels of light PA [31], total PA [32] and MVPA [34] than students in traditional classrooms.*

5. Comparative Discussion: The discussion section could be enhanced by comparing the results with other types of interventions aimed at increasing physical activity levels. This could include exploring whether other interventions lead to different outcomes and analyzing the potential causes for the observed effects in ALCs.

**Author’s response:** We thank the reviewer for his insightful comment. Following the suggestion, we have added a paragraph in the discussion section to better address the results regarding the effect of ALCs on increasing PA levels.

*A possible explanation for the differences in the effect of interventions on increasing PA observed in this review may lie not so much in their design but in the use and dynamics established for working in these spaces. Interventions that implement zone-based routines [17] or specific methodologies promoting student-centered learning while optimizing the space [16] appear to have been more effective than those that did not specify such practices [31, 32]. Class size may also have been a key factor, as larger classrooms might encourage greater PA [15, 16].*
