# Supplementary material for: Impact and perceptions of Active Learning Classrooms on reducing sedentary behaviour and improving physical and mental health and academic indicators in children and adolescents: A scoping review
Source: PLoS One. 2025 Feb 5;20(2):e0317973. doi: 10.1371/journal.pone.0317973 (PMC11798443; doi:10.1371/journal.pone.0317973)
Supplement: Attachment — Submitted filename: Response to Reviewers.docx [file pone.0317973.s005.docx]

Heather Macdonald, Ph.D

Academic Editor

PLOS ONE

Dear Editor,

Please find enclosed a revised version of our manuscript, “Impact and perceptions of Active Learning Classrooms on reducing sedentary behaviour and improving physical and mental health and academic indicators in children and adolescents: a scoping review” (**PONE-D-24-40271R1**). Once again, we would like to thank you for the opportunity to review and improve our manuscript and the reviewers for their thoughtful and constructive comments. We have taken all suggestions into account and incorporated them into the revised manuscript ('Revised Manuscript with Track Changes') and believe that our manuscript is stronger as a result. Below is a point-by-point response to reviewer 1's comments.

Thank you for your attention to this matter.

**Response to Journal Requirements:**

Please review your reference list to ensure that it is complete and correct. If you have cited papers that have been retracted, please include the rationale for doing so in the manuscript text, or remove these references and replace them with relevant current references. Any changes to the reference list should be mentioned in the rebuttal letter that accompanies your revised manuscript. If you need to cite a retracted article, indicate the article’s retracted status in the References list and also include a citation and full reference for the retraction notice.

**Author’s response:** The reference list has been carefully reviewed and verified to ensure that it is complete and correct. Thank you.

**Review Comments to the Author**

**Reviewer #1:** Dear Authors,

After reading the revised manuscript, I believe the revisions have improved it. I am satisfied with your revisions to my previous comments. I also think that the revised objectives helped clarify the intentions of the review. I have a few detailed comments, and I wish you good luck finalising the manuscript.

Detailed comments:

Lines 32-35. I find the wording a bit awkward. It is easier to write ‘…learning spaces (ALCs) impacts movement patterns, physical or mental health…’ or something similar.

**Author’s response:** Thank you to the reviewer for your valuable comment. We have revised the sentence to make it simpler and more straightforward, as suggested.

*“…We searched for peer-reviewed quantitative and qualitative studies published in English that examined the impact of ALCs on movement patterns, physical or mental health, and academic indicators in children and adolescents, as well as those that explored the perceptions of members of the educational community and the teaching practices used in ALCs…”*

Lines 37-38. Please change ‘Eighteen studies’ to Nineteen studies and adjust the count regarding study designs.

**Author’s response:** Done. Thank you.

Line 107. For clarity, you might consider changing ‘these learning spaces’ to ALCs. A change here also involves a change in the Abstract.

**Author’s response:** Changed in both sections.

Lines 215-216. I recommend placing Australia before Finland and Belgium before Israel to follow alphabetic order when describing the number of included studies.

**Author’s response:** Done. Thanks for the recommendation.

Line 240. I think the reference [28] should be included at the end of the sentence.

**Author’s response:** It is true. We have added it. Thank you very much.

Line 365. I recommend that you write ‘The size of classrooms…’ as class size is potentially misleading and might be interpreted as the number of students in each class.

**Author’s response:** We completely agree. We have changed it. Thank you for your comments.

Finally, I recommend that the authors carefully proofread the manuscript.

**Author’s response:** The reviewer’s valuable comment is appreciated, as well as their dedication to the review process. The manuscript has been carefully proofread to ensure clarity and accuracy.

**Reviewer #2:** I have reviewed the revised manuscript titled "Impact and perceptions of Active Learning Classrooms on reducing sedentary behaviour and improving physical and mental health and academic indicators in children and adolescents: a scoping review.

I am pleased to report that the authors have adequately addressed the concerns and suggestions raised in my previous review. The revisions have strengthened the manuscript, and I believe it now meets the publication standards of PLOS ONE.
**Author’s response:** We appreciate the reviewer's comment.
