# Supplementary material for: Impact and perceptions of Active Learning Classrooms on reducing sedentary behaviour and improving physical and mental health and academic indicators in children and adolescents: A scoping review
Source: PLoS One. 2025 Feb 5;20(2):e0317973. doi: 10.1371/journal.pone.0317973 (PMC11798443; doi:10.1371/journal.pone.0317973)
Supplement: Attachment — Submitted filename: Response to Reviewers.docx [file pone.0317973.s006.docx]

Heather Macdonald, Ph.D

Academic Editor

PLOS ONE

Dear Editor,

Please find enclosed a revised version of our manuscript, “Impact and perceptions of Active Learning Classrooms on reducing sedentary behaviour and improving physical and mental health and academic indicators in children and adolescents: a scoping review” (**PONE-D-24-40271R2**). Once again, we would like to thank you for the opportunity to review and improve our manuscript. We have taken all suggestions into account and incorporated them into the revised manuscript ('Revised Manuscript with Track Changes'). Below is a point-by-point response to comments.

Thank you for your attention to this matter.

**Response to Journal Requirements:**

Please review your reference list to ensure that it is complete and correct. If you have cited papers that have been retracted, please include the rationale for doing so in the manuscript text, or remove these references and replace them with relevant current references. Any changes to the reference list should be mentioned in the rebuttal letter that accompanies your revised manuscript. If you need to cite a retracted article, indicate the article’s retracted status in the References list and also include a citation and full reference for the retraction notice.

**Author’s response:** The reference list has been carefully reviewed and verified to ensure that it is complete and correct. Thank you.

**Response to additional Editor Comments:**

Thank you for addressing the 2nd round of comments. Although the authors noted that they proofread the manuscript in response to the reviewer's request, I note a few final typographical/grammatical errors that should be corrected. Please note that PLOS One does not copyedit accepted manuscripts before publication so the authors may wish to consider professional language and/or copy editing services.

**Author’s response:** The editor’s valuable comment is appreciated, as well as their dedication to the review process.

1. Spelling of behavior/behaviour is inconsistent throughout the manuscript. Please use Find/Replace to use one spelling throughout.

**Author’s response**: Done, except in the references section where we have kept the word in American English to respect the titles of the journals where they are published. Thank you.

2. Line 38: Add "children's" before sedentary

**Author’s response:** Done

3. Line 61: Remove "the" before the percentage range.

**Author’s response:** Done

4. Line 90: Does "none of them" refer to the 2 reviews mentioned above? If so, change to "neither review", and use a comma to join the following sentence (...mental health, nor did these reviews answer...)

**Author’s response:** Yes, we have made the suggested changes.

5. Line 196: Change "of them" to "studies" - same on line 202 - change "of them" to "studies"

**Author’s response:** Done

6. The text in the 4th column of Table 2 appears to be right justified and this format isn't used in the other tables - please modify

**Author’s response:** Done

7. Line 288: Change describe to described

**Author’s response:** Done

8. Line 291: Change include to included

**Author’s response:** Done

9. Line 308: Please modify the sentence so it doesn't start with "And"

**Author’s response:** Done

10. Line 314: Add "included" before studies. Also, "but sometimes" seems rather vague and informal

**Author’s response:** Done. We have rephrased the phrase as follows:

*“Overall, most of the included studies showed an increase in standing time and a decrease in sitting time, but at the same time no change in step count, step time, or PA”*

11. Line 323: Consider adding "physical" before size.

**Author’s response:** Done. Thanks for the recommendation.

12. Line 331: Consider changing to "...students in ALCs"

**Author’s response:** We agree. Done. Thank you for your comments.

13. Line 334: Please correct the grammar in this sentence as "...reported in other study in university students" is poorly worded

**Author’s response:** Done

14. Line 338: "...different furniture that allows not only to vary..." does not make sense - please modify

**Author’s response:** Done. We have rephrased the phrase as follows: *“…, as well as diverse furniture that allows not only for a transition from sitting to standing but also for alternating sitting postures, which could explain the...”*

15. Line 350: Did this study actually assess the social construct of gender? If not, please change this to sex differences

**Author’s response:** Thank you for this appreciation; it refers to sex differences. We have changed it. Thanks.

16. Line 353: Remove "in" before "which"

**Author’s response:** Done

17. Line 359: Change to "teachers described distraction as a barrier for some students"

**Author’s response:** Done

18. Line 361: Add "the" before Kariipanon

**Author’s response:** Done

19. Line 362: Should "active learning spaces" be changed to ALCs?

**Author’s response:** Yes, that is right. Changed. Thank you

20. Line 365: Reference 9 cited here has round brackets whereas other references are in square brackets

**Author’s response:** Changed. Thank you

21. Line 369: Change "as self-reported" to "by self-report"

**Author’s response:** Done

22. Line 370: Remove "would"

**Author’s response:** Done

23. Line 382: Remove "use" after ALCs

**Author’s response:** Done

24. Line 389: Consider changing "their findings" to "study findings" or just say "findings"

**Author’s response:** Thank you for your comment. We have decided to only leave “findings”

25. Line 402: Change "ALCs based interventions" to ALC interventions

**Author’s response:** Done

26. Line 405: Change "four" to 4

**Author’s response:** Done

27. Line 409: Consider adding "children's" before sedentary

**Author’s response:** Done. We have added “Children´s”.
